# Robotic subxiphoid-optical thymectomy

**DOI:** 10.1093/icvts/ivac104

**Published:** 2022-04-21

**Authors:** Masanori Shimomura, Shunta Ishihara, Satoru Okada, Masayoshi Inoue

**Affiliations:** Division of Thoracic Surgery, Department of Surgery, Kyoto Prefectural University of Medicine, Kyoto, Japan

**Keywords:** Robotic surgery, Subxiphoid approach, Thymectomy

## Abstract

Robot-assisted thymectomy through a subxiphoid scopic approach can provide a good surgical view, similar to that of median sternotomy. We originally used the subxiphoid port only for the robotic scope to avoid instrument collision with the assistant device. This approach, robotic subxiphoid-optical thymectomy, is advantageous for the safe and accurate dissection of the bilateral phrenic nerves and the left brachiocephalic vein, which are especially needed in extended thymectomy for patients with myasthenia gravis.

## INTRODUCTION

Robotic thymectomy for thymic tumour and myasthenia gravis (MG) show good perioperative results and low local recurrence with med-term outcomes [1]. Trans-subxiphoid robotic thymectomy (TSRT) recently has the advantage of cosmetic aspects and good views around the left phrenic nerve and the upper pole of the thymus with uniportal assistance [2] or without port assistance [3].

We herein introduce a modified TSRT procedure as robotic subxiphoid-optical thymectomy (RST). The originality of RST is its use of only the robotic scope through the subxiphoid incision to avoid collision between the assistant device and the robotic arm.

## TECHNIQUE

**Figure 1:**
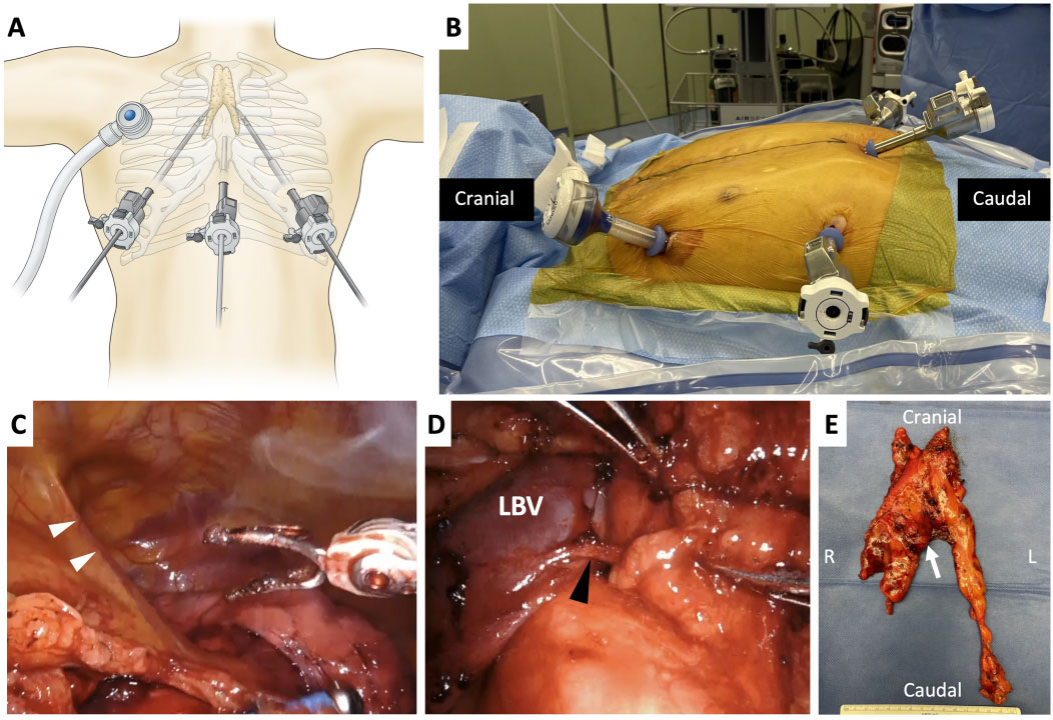
(**A**) Schema of robotic subxiphoid-optical thymectomy. (**B**) Port placement. (**C**) Dissecting the thymus from the left phrenic nerve (white arrowhead). (**D**) Dissecting the left brachiocephalic vein and thymic vein (black arrowhead). (**E**) Surgical specimen for robotic subxiphoid thymectomy, white arrow; tumour.

The operation is performed under general anaesthesia with differential lung ventilation using a double-lumen tube. The patient is positioned supine with a cushion on the right back and the right upper limb in a mild extended and dropped position. We used the Da Vinci Xi™ surgical system (Intuitive Surgical, Sunnyvale, CA, USA) with 3 arms: 8-mm ports placed in the right 6th intercostal space on the midclavicular line (arm 1), subxiphoid (arm 2: Camera, 30° oblique view) and the left 6th intercostal space on the midclavicular line (arm 3). A 12-mm Air Seal™ port (Conmed, Milford, CT, USA) is placed on the anterior axillary line of the right third intercostal space as an assist port (Fig. 1A and B). The right thoracic cavity is first observed through the assist port followed by the insertion of robotic arm-1 and arm-2 ports. The mediastinal pleura is opened to reach the left thoracic cavity, and the operation can be completed under bilateral ventilation using CO_2_ insufflation. The arm-3 port is placed, and the patient cart of the Da Vinci Xi™ surgical system is rolled in and docked. The Long Bipolar Grasper™ and Bipolar Maryland Forcep™ (Intuitive Surgical) are used for both hands. The thymic tissue is dissected from the pericardium along with the identification of the right and left phrenic nerves, and the dissection proceeds towards the cranial side of the anterior mediastinum (Fig. 1C). The right and left brachiocephalic veins are identified, followed by the superior vena cava, and further dissection is performed to the left along the left brachiocephalic vein, which could be the pitfall of accidental bleeding during thoracoscopic thymectomy. Several thymic veins are dissected using the Vessel Sealer Extended™ (Intuitive Surgical) or an energy device of LigaSure Maryland™ (Medtronic, Minneapolis, MN, USA) by the assistant (Fig. 1D). The specimen is extirpated through the assist port (Fig. 1E), followed by the insertion of a 19-Fr multichannel slit drain through the subxiphoid incision (Video 1).

We performed RST in 10 cases between October 2020 and December 2021. The procedure was extended thymectomy for MG or thymomatous MG in 2 patients and total thymectomy for thymic tumours in 8 patients (Table 1). The mean console time was 134 (83–210) min, and the mean time to roll-in was 37 (26–55) min. There were no conversions to thoracotomy and no complications for Clavien–Dindo grade IIIA or higher than 90 days after surgery in any case. Two patients who underwent MG thymectomy had no MG crisis and were in remission for 1 year and 6 months after surgery, respectively. The clinical ethics committee of Kyoto Prefectural University of Medicine (Kyoto, Japan) approved this clinical study (ERB-C-1387-2), and the opt-out policy for this study was available on the homepage of Kyoto Prefectural University of Medicine instead of obtaining informed consent from all participants.

**Table 1:** Robotic subxiphoid-optical thymectomy in 10 cases

Diseases	*N*
Thymoma	3
Thymomatous MG	1
Stage^a^	
I	2
II	2
III–IV	0
MG	1
Lymphoma	1
Thymic cyst	2
Thymic hyperplasia	2

a Stage on 8th edition of TNM classification.

MG: myasthenia gravis.

## DISCUSSION

In the present RST procedure, the surgical view is similar to that of median sternotomy, and safe dissection around the phrenic nerves is possible, even in patients with thymic tumours. The lateral approach, which is currently major, is often used for robot-assisted endoscopic thymectomy for thymic tumours and MG to allow easy recognition of mediastinal anatomy from the thoracic cavity [4]. It, however, might occasionally be insufficient to detect the opposite side of the phrenic nerve and of the upper polar of the thymus. We also had the same experiences in the right lateral approach. TSRT is another optional procedure of robotic thymectomy, in which the assist port is placed neighbour to the robotic scope under the xiphoid while reducing the number of ports [2]. However, intraoperative collision with the robotic scope can cause limited manipulation of the assistant, especially during dissection of the upper pole of the thymus. With RST, the assistant port located distant from the robotic scope or instruments can avoid instrument collision and allow safe vascular dissection using energy devices by the assistant. Unfortunately, it is not possible to compare the RST with the lateral approach using the data.

We have not yet evaluated postoperative pain for this procedure with multiple ports due to the small sample size. We need to accumulate more cases to evaluate whether RST is a patient-friendly technique in thymectomy with its long-term outcome of MG or thymoma.

In conclusion, the RST could be a safe and useful approach for total thymectomy, which enables comfortable cooperation by the console surgeon and assistant.

**Conflict of interest:** none declared.

### Reviewer information

Interactive CardioVascular and Thoracic Surgery thanks Clemens Aigner, Rui Haddad and the other, anonymous reviewer(s) for their contribution to the peer review process of this article.
